# Nod-like receptors in inflammatory arthritis

**DOI:** 10.1016/j.bj.2023.100655

**Published:** 2023-08-19

**Authors:** Sahib Singh Madahar, Alita Gideon, Ali A. Abdul-Sater

**Affiliations:** a School of Kinesiology and Health Science, Muscle Health Research Centre, York University, Toronto, Ontario, Canada; b Department of Biology, York University, Toronto, Ontario, Canada

**Keywords:** Nod-like receptors, Rheumatoid arthritis, Psoriatic arthritis, Gout, Ankylosing spondylitis, Pediatric arthritis

## Abstract

Nod-like receptors (NLRs) are innate immune receptors that play a key role in sensing components from pathogens and from damaged cells or organelles. NLRs form signaling complexes that can lead to activation of transcription factors or effector caspases – by means of inflammasome activation –Inflammatory arthritis (IA) culminating in promoting inflammation. An increasing body of research supports the role of NLRs in driving pathogenesis of IA, a collection of diseases that include rheumatoid arthritis (RA), psoriatic arthritis (PsA), ankylosing spondylitis, and pediatric arthritis. In this review, we briefly discuss the main drivers of IA diseases and dive into the evidence for – and against – various NLRs in driving these diseases. We also review the studies examining the use of NLR and inflammasome inhibitors as potential therapies for IA.

## Highlights

• Nod-like receptors (NLRs) are a family of intracellular innate immune receptors.
• Some NLRs play a key role in pathogenesis of various inflammatory arthritis (IA).
• Natural, synthetic and dietary compounds targeting NLRs can attenuate inflammation in models of IA.

## Introduction

### NOD-like receptors protein structure, function, and signal transduction

Nucleotide-binding and oligomerization domain (NOD)-like receptors (NLRs) are a cytosolic family of intracellular pattern recognition receptors (PRRs) that play a key role in the innate immune response to pathogens and other types of cellular stress. These receptors are expressed in various immune cells, including dendritic cells, lymphocytes, neutrophils, monocytes, and macrophages. They are composed of a C-terminal leucine-rich repeat (LRR), a central nucleotide-binding domain (NBD) and a N-terminal effector domain, that binds to adaptor molecules for downstream signaling. Dysregulation of NLRs has been linked to several inflammatory and autoimmune disorders, including rheumatoid arthritis (RA), Crohn's disease, and type 2 diabetes [1]. Several NLR family members are key components of the multiprotein complex, known as inflammasome, which leads to activation of caspase-1 and triggers the release of pro-inflammatory cytokines such as interleukin 1β (IL-1β) and interleukin-18 (IL-18) via gasdermin D (GSDMD) mediated pores in the plasma membrane. This ultimately leads to pyroptosis, a form of programmed cell death [2].

There are five subfamilies of NLRs: NLRA, NLRB, NLRC, NLRP and NLRX [3]. Of these, the NLRC and NLRP subfamilies comprise the most members and are the most studied. The largest NLR subfamily is made up 14 members (NLRP 1–14). This subfamily is known as NLRP because it contains a pyrin domain (PYD) at the N-terminus. Of these, NLRP3 is the best studied and can recognize danger-associated molecular pattern molecules (DAMPs; e.g., adenosine triphosphate-ATP, hyaluronic acid, uric acid, monosodium urate- MSU crystals and cholesterol crystals), and different environmental factors (e.g., silicon, aluminum, asbestos, skin irritants, ultraviolet-UV radiation). NLRP3 can also recognize a wide range of secreted microbial toxins, and components of viruses, fungus, bacterial or parasites [4]. The second largest class, NLRC, is made up of 5 members (NLRC 1–5). These proteins contain the N-terminal caspase activation and recruitment domain (CARD). The first NLRs to be identified are NOD1/NLRC1 (CARD4) and NOD2/NLRC2 (CARD2), which are considered essential, or major receptors, as they play a key role in the activation of nuclear-factor kappa-light-chain-enhancer of activated B cells (NF-kB), a master transcription factor [5]. They recognize conserved motifs in bacterial peptidoglycan [5]. NLRC4 are critical for activating caspase-1 in response to bacterial T3SS and flagellin, but it requires cooperation with neuronal apoptosis inhibitory protein(NAIP) proteins, which can directly sense the ligand [6]. Conversely, NLRC3 has been shown to modulate innate immune responses by inhibiting NF-κB and stimulator of interferon genes tank-binding kinase 1 (STING/TANK1) signaling pathways [7].

### The inflammasome

Inflammasomes are multimeric protein complexes, typically made up of 3 components: a DAMP/PAMP pathogen-associated molecular pattern (PAMP) sensor (e.g., NLR or Aim2-like recptor-ALR), the apoptosis-associated speck-like protein containing a CARD (ASC) adaptor protein, and the effector enzyme, caspase-1 [4]. Of the NLR-forming inflammasomes, NLRP1, 2, 3, 6, 7, 12 and NLRC4 (after activation by NAIP) are involved in inflammasome activation, but the NLRP3 inflammasome is the most well studied amongst them [2,8]. The role of the NLRP3 inflammasome was first defined by the discovery of a gain of function mutation in NLRP3, leading to cryopyrin-associated periodic syndrome (CAPS). This mutation causes abnormal inflammasome activation and downstream inflammatory signaling [9].

To activate the inflammasome, two signals are usually required: the priming signal and the activation signal. The priming signal (signal 1) is triggered by the activation of innate immune receptors to PAMPs, sterile activators (DAMPs), lipopolysaccharides (LPS) or phorbol myristate acetate (PMA). This allows the activation of the transcription factor NF-kB and expression of precursor proteins and components (NLRs, pro-IL-1β and pro-caspase-1) required for the inflammasome complex. Signal 2, or activation signal, is triggered by a myriad of damaging molecules (e.g., MSU crystals, extracellular ATP or bacterial toxins like nigericin), which drives post translational aggregation and polymerization of the inflammasome components ultimately leading to cleavage of pro-caspase-1 to active caspase-1. Caspase-1 will then cleave pro-IL-1β and pro-IL-18 into their biologically active forms, IL-1β and IL-18 [4]. Once cleaved, these potent pro-inflammatory cytokines can be released from the cell via GSDMD pores in the plasma membrane formed by N-terminal GSDMD fragments (GSDMD-N) following cleavage by caspase-1 [2].

#### Inflammatory arthritis

Inflammatory arthritis (IA) is a collection of diseases that involves the manifestation of most classical symptoms of inflammation that eventually lead to pain, swelling and stiffness of the joint as a result of an overactive immune system [10]. The common subtypes of IA are RA, psoriatic arthritis (PsA), and ankylosing spondylitis (AS). Individuals of any age, ethnicity and sex can be affected by IA although susceptibility of certain groups are higher depending on the type of IA disease [11].

Hider and colleagues (2019) provided a questionnaire to UK primary care patients seeking help for musculoskeletal (MSK) and non-musculoskeletal (non-MSK) concerns to better understand how symptoms are reported [10]. Interestingly but not surprisingly, non-MSK patients reported persistent joint pain, swelling and stiffness, symptoms that are otherwise predictive of IA. Patients with early-stage RA present with morning stiffness, fatigue, fever, weight loss, swollen and tender joints, rheumatoid nodules under the skin, elevated levels of C-reactive protein (CRP) and increased erythrocyte sedimentation rate (ESR). Clinically, RA affected areas are found in proximal interphalangeal (MIP) and metacarpophalangeal (MP) joints [12]. Chronic inflammation in RA can contribute to serious systemic manifestations including atherosclerosis, bone erosion, cartilage destruction, lymphomas, hematologic abnormalities, loss of range of motion and joint malalignment [12]. MSK features of PsA have been divided into two categories, namely, peripheral and axial manifestations. The heterogeneity of this disease has been characterized and divided into five patterns: polyarticular, oligoarticular, distal, arthritis mutilans, and axial or spondyloarthritis [13]. Systemic juvenile idiopathic arthritis (JIA) has been characterized by a recurrent rash and fever while oligoarticular JIA has been associated with bone erosion and uveitis [14].

Like many diseases, there are many factors that can increase the risk of developing IA, including age, environment, and genetics (reviewed in Ref. [15]). It is important to recognize RA manifestation early and begin treatment immediately. Several studies have suggested that early treatment can contribute to reduced disease outcome [16]. DNA methyltransferases (DNMTs), histone acetyltransferases (HATs) and histone deacetylases (HDACs) could serve as potential regulators of pathogenesis by remodelling the epigenetic modifications on the chromatin [17]. Givinostat (ITF 2357) is a Histone deacetylase inhibitor (HDACi) used in treating systemic onset JIA and is reported to have significant benefits after 12 weeks of treatment [17]. This HDACi is also responsible for reducing inflammatory cytokines in RA fibroblast-like synoviocytes (FLS), which otherwise contribute to inflammation in and destruction of the joint [17,18]. The mechanism by which HDACi supress inflammation in RA is through inhibiting IL-6 mRNA stability in FLS and macrophages and promoting mRNA decay [18]. Etanercept is the first approved biologic that inhibits tumor necrosis factor (TNF) for the treatment of RA; by delaying the onset and reducing the symptoms, it improves the quality of life [19]. Glucocorticoids treatment greater than 5 mg/dl for RA patients is associated with an increased mortality risk in a dose-dependent manner as well as an increased risk of cardiovascular events [20]. Listing and colleagues (2015) observed in their study that TNFα inhibitors and rituximab reduce the mortality rate compared to treatments with methotrexate only or in combination with synthetic disease-modifying antirheumatic drugs (DMARDs) [20]. DMARDs are divided into three categories: (1) biologic DMARDs (i.e., co-stimulatory molecule inhibitors, IL-6 inhibitors, TNF-α inhibitors); (2) conventional synthetic DMARDs (i.e., hydrochloroquine, methotrexate and sulfadiazine) (3) targeted synthetic DMARDs (i.e., Janus kinase-JAK inhibitors); [21]. For patients unresponsive to DMARDs, an alternative therapy of tocilizumab only (a non-TNF biological agent) or in combination with methotrexate is recommended [22].

The overall objective of this review is to discuss the studies elucidating the role of NLRs in the pathogenesis and progression of various types of IAs, describe the contribution of different immune cells in NLR-mediated inflammation, and summarize current therapeutic approaches.

## Methodologies

The searches in this review were conducted in the MEDLINE database using several keywords related to the role of NLRs in IA. The following MeSH terms were used to identify studies to include in the review: nod-like receptors, inflammasomes, IA, RA, PsA, gout, AS, pediatric arthritis, juvenile idiopathic arthritis and juvenile spondylarthritis. The search criteria were also applied to studies that were found in the reference lists of the included papers. Additional studies summarizing previously reviewed materials were included in this review if they were relevant to the search criteria in order to highlight and connect key concepts.

## NOD-like receptors in gout

Gout (or crystal arthritis) is an IA that arises due to hyperuricemia (serum urate levels >7 mg/mL) [23]. This increased concentration causes monosodium crystal deposition at the joints (usually in a single joint), which triggers acute episodes of joint inflammation. Gout can also be caused by calcium pyrophosphate dehydrate (CPPD) crystals deposition in the joints. If left untreated, tophi begin forming in the soft tissue resulting in bone damage and chronic flare ups. There are four distinct stages to the disease: 1) Patients experience higher serum urate than physiologically normal, 2) urate crystals form in the joints, 3) deposited crystals are sensed by resident macrophages leading to activation of NLRP3 inflammasomes and secretion of IL-1β, and 4) involvement of innate and adaptive immune cells and with bone and tissue damage becoming visible [24]. There are a multitude of pathways that regulate the activity of the inflammasome and the subsequent release of IL-1β. Some strategies to treat the disease look at targeting crystal formation, lowering uric acid concentrations in the blood, and mediating the body's inflammatory response.

Unlike other autoinflammatory diseases, gout is not genetically driven. Rather, it is caused by the activation of the innate immune system in response to MSU crystals, an endogenous substance, released by damaged cells, that is not considered to be proinflammatory in nature [23]. MSU crystals activate innate cells in the joint, serving as signal 2, triggering NLRP3 inflammasome assembly and downstream release of mature IL-1β [8]. Subsequently, the release of IL-1β drives acute inflammation and recruitment of neutrophils, monocytes and other immune cells to the affected area, giving rise to joint swelling and severe pain [23].

There are several *in vitro* and *in vivo* studies demonstrating the role of NLRP3 in gout. Most notably, Martinon et al. (2006) were the first to show that MSU crystals trigger NLRP3 inflammasome activation and that the addition of a caspase-1 inhibitor, zYVAD-fmk, blocked the release of mature IL-1β [25]. Mice with macrophages deficient in caspase-1, ASC or NLRP3 were defective in MSU crystal-induced IL-1β secretion [25]. Intriguingly, a new study revealed that Tumor necrosis factor receptor (TNFR)-associated factor 1 (TRAF1), an immune signaling adapter involved in regulation of NF-κB activation, negatively regulates inflammasome activation and limits joint swelling and inflammation in a model of MSU-crystal induced gout [26]. Moreover, several studies have found that certain NLRP3 polymorphisms, most notably rs3806268 and rs10754558, were associated with an increased susceptibility to gout [27,28].

Given the well-documented role of NLRP3 in driving gout pathogenesis, a plethora of studies focused on identifying natural products, dietary elements, and novel compounds that can be used to target NLRP3 inflammasomes in gout, as well as elucidating their mechanism of action (reviewed in Ref. [29]). All of these studies demonstrate the role of the NLRP3 inflammasome and how its downstream signaling affects the development of gout and its symptoms. Cucurbitacin (CuB), a tetracyclic triterpene isolated from plants, was shown to reduce foot swelling and inflammatory cell infiltration in a model of MSU-induced gouty arthritis in mice [30]. The authors also showed that CuB can specifically inhibit the inflammasome, as treatment with CuB reduced *NLRP3* mRNA and protein and inhibited inflammasome formation in murine bone marrow derived macrophages (BMDMs) [30]. Another group looked at the effects of Celastrol, a pentacyclic triterpenoid used in traditional Chinese medicine, and showed that it alleviated MSU-induced gouty arthritis in mice by inhibiting NLRP3 inflammasome assembly by blocking BRCA1-BRCA2-containing complex subunit 3 (BRCC3) mediated K63 deubiquitination of NLRP3 [31]. Palmatine [32], tetrahydropalmatine (THP) [33], gallic acid [33], and epigallocatechin gallate (EGCG) [34], have all also been shown to attenuate MSU-crystal gouty arthritis by reducing oxidative stress-induced NLRP3 inflammasome activation. Oridonin, a major active ingredient of the traditional Chinese medicinal herb *Rabdosia rubescens*, covalently binds to cysteine 279 of NLRP3 and blocks its interaction with NEK7 to limit gouty arthritis [35]. Overall, these findings reveal the promising effects of naturally derived compounds in inhibiting NLRP3 activation and ameliorating gout.

Novel compounds targeting NLRP3 have also been investigated as a target to reduce inflammasome activation in gout. Inhibitors are becoming increasingly relevant in aiding our understanding of the underlying mechanism of gout, while providing hope to people living with the disease. In murine arthritic models, the NLRP3 inflammasome inhibitor OLT1177 (safe for use in humans), limited MSU-crystal inflammation, which was associated with decreased synovial IL-1β and IL-6 levels [36]. Additionally, 2-phenyl-benzoxazole acetamide derivative (compound 52) [37], (E)-1-(6-methoxybenzo [d]oxazol-2-yl)-2-(4-methoxyphenyl)ethanone oxime (5d) [38], NSC697923 [39], and the curcumin analogue, AI-44 [40], have all been tested in MSU-crystal gout models and shown promise in reducing joint and paw inflammation. Interestingly, Coll and colleagues (2015) described a small-molecule inhibitor, sulfonylurea (MCC950), that potently blocks NLRP3 activation with high specificity and reduced IL-1β secretion in response to MSU-crystal induction [41]. However, whether this inhibitor can be used to limit gout has not yet been explored.

Dietary compounds like omega-3 fatty acids can benefit inflammation-driven diseases and has potential clinical use in autoinflammatory and NLRP3-inflammasome-driven diseases, like gout. BMDMs primed with LPS, incubated with omega-3 fatty acids and later stimulated with MSU specifically inhibited NLRP3 and NLRP1 inflammasome activation and subsequent IL-1β production, by suppressing caspase-1 activity [42]. Recently, investigating the relationship between the ketogenic diet and inflammation has gained popularity. The ketogenic diet increases B-hydroxybutyrate (BHB), which has been associated with reduced inflammatory effects [43]. Increased BHB relieved MSU-induced gout in mice, without affecting defence against bacterial infection. BHB inhibited NLRP3 inflammasome in S100A9 primed and MSU activated macrophages and reduced IL-1β secretion from neutrophils [44]. All these studies revealed an interesting interplay between metabolism and inflammatory driven diseases, like gout.

Although NLRP3 has been extensively linked to pathogenesis of gout, other NLRs may also contribute to this disease. Recently, it was noted that NAIP1 may be involved in gout pathogenesis [45]. Murine NAIP1, expressed in human macrophages, induced IL-1β and downstream inflammatory signaling in response to soluble uric acid. The authors also demonstrated that NAIP1 directly recognizes soluble uric acid, being the first to report that NAIP may recognize a DAMP [45].

Together, all these findings demonstrate the complex signaling pathways that drive gouty arthritis. Interestingly, the mechanosensitive Transient Receptor Potential Vanilloid 4 (TRPV4) channel, which is highly expressed by synovial macrophages of mice injected with MSU-crystals and peripheral blood mononuclear cells (PBMCs) from gout patients, may mediate NLRP3 inflammasome activation by endogenous and exogenous crystalline materials, including MSU crystals [46]. However, it is still unclear what the precise mechanism of MSU crystal recognition is, and whether other NLR family members may play a role in this process and in driving gout pathogenesis.

## NOD-like receptors in rheumatoid arthritis

RA is a chronic and complex inflammatory disease that is characterized by persistent and excessive inflammation of the synovial joint leading to the destruction of cartilage and bone [47]. RA pathogenesis involves a wide array of immune cells and cytokines such as IL-1, IL-6, IL-18 and TNF [47].

In addition to Human leukocyte antigen (HLA)-DRB1 gene, protein tyrosine phosphatase non-receptor 22 (PTPN22) gene polymorphisms are highly associated with increased susceptibility and severity of RA [48]. NLRP3 inflammasome has been recognized for its role in producing IL-1 and IL-18 which suggests that it has a role in RA pathogenesis [49]. Spalinger and colleagues (2016) discovered that PTPN22 positively regulates NLRP3 inflammasome activation by dephosphorylating the Y861 residue on NLRP3 [50]. Interestingly, PTPN22 variants have been implicated in numerous inflammatory disorders including RA [51,52]. The PTPN22 variant rs2476601 (R620W), considered gain-of-function, has enhanced dephosphorylation activity which promotes NLRP3 inflammasome activation thereby increasing IL-1β secretion [50]. Similarly, mice deficient in PTPN22 had decreased NLRP3 activation and less secretion of IL-1β. Begovich and colleagues (2004) reported that the R620W missense in PTPN22 disrupts the P1 proline-rich motif which interacts with Csk; this disruption impairs the role of these proteins as negative regulators of T-cell activation [51]. On the contrary, some studies report that R620W produces a gain-of-function inhibition of T and B cell activation as a result of the increased phosphatase activity in T cell receptor (TCR) and B cell receptor (BCR) signalling. Autoimmunity because of decreased immune cell activation appears counterintuitive. However, a few models have been proposed, one being that decreasing TCR signalling at the thymic level leads to less negative selection and elimination of autoreactive T cells and a reduced production of natural regulatory T cells (Treg) [53,54]. It is important to note that there is conflicting information in literature about the role of the R620W variant in RA susceptibility. The R620W polymorphism has been identified as a risk factor for RA in several population studies [[55], [56], [57], [58]]. Notably, the R620W variant enhances neutrophil effector functions in both healthy and RA patients, however, since neutrophils are abundant in the RA joint, increased neutrophil migration, Ca2+ release and reactive oxygen species (ROS) production can contribute to joint damage and initiate the disease-associated inflammation [59]. On the other hand, a population study by Tang and colleagues (2016) that established several associations between PTPN22 polymorphism did not support a link between the R620W variant and RA susceptibility [60]. Another PTPN22 variant rs33996649 (R263Q) has been recognized for its protective role against RA as this missense occurs in the catalytic domain which reduces its phosphatase activity [53,61,62]. Another population study by Yang and colleagues (2015) identified several polymorphisms in secreted protein acidic and rich in cysteine (SPARC) and NLRP2 that are associated with RA susceptibility [63].

Zhang et al. (2016) employed the collagen-induced arthritis (CIA) model to investigate the role of NLRP3 expression in the development of arthritis. They showed that during the early onset of RA, there is an increased expression of NLRP3 and that the levels directly correlated with disease severity [64]. Interestingly, another group reported a high activation of NLRP3 inflammasome in synovia from both RA patients and CIA mice [65]. In this study, Guo and colleagues (2018) administered MCC950, a highly specific NLRP3 inhibitor, to mice with CIA and found significantly less inflammation of joints and reduced bone destruction [65]. In a spontaneous polyarthritis model, A20^Myel−KO^, a myeloid-cell specific deletion of an RA susceptibility gene A20/Tnfaip3 contributed to an enhanced NLRP3 driven caspase-1 activation, pyroptosis, and IL-1β release [66]. Deletion of NLRP3, caspase-1 or IL-1 receptor was responsible for rescuing the RA-associated inflammation and cartilage destruction in these mice [66]. Choulaki and colleagues (2015) found that whole blood cell samples from human RA patients stimulated through TLR3 and TLR4 but not TLR2 had increased expression of NLRP3 and higher levels of IL-1β that was driven by caspase-1 and caspase-8 when compared to healthy controls [67]. On the contrary, an earlier study by Kolly and et al. (2009) employed an antigen-induced arthritis (AIA) model and showed that while ASC is responsible for RA pathogenesis, ASC-associated inflammation is modulated independently of other inflammasome components such as caspase-1, NLRP3 and NLRC4 [68].

In addition to NLRP3, a few studies have investigated the role of other NLR proteins in RA. A study by Liu and colleagues (2013) determined that NLRC5 regulates the NF-κB signalling pathway to promote cell proliferation and secretion of inflammatory cytokines of FLS to contribute to RA progression [69]. Interestingly, attenuated expression of NLRP6, which is known for its role as a negative regulator of intestinal inflammation [70], is found in the synovial tissues and FLS of RA patients compared to osteoarthritis (OA). In particular, low NLRP6 expression is associated with a weak interaction between tripartite motif containing 38 (TRIM38) and TGF-β activated kinase 1 binding protein 2/3 (TAB2/3), resulting in increased levels of TAB2/3 leading to increased NF-κB activation mediated inflammation [71]. This study opens the possibility for novel therapies targeting NLRP6 for RA treatment.

Outside of innate immune cells, NLRP3 inflammasome activation contributes to autoimmune disease activity in RA patients as it promotes Th17 cell differentiation, which, in turn, is correlated with increased levels of IL-1β and IL-17 in sera [72]. The same study reported that the use of caspase-1 or IL-1 receptor inhibitors, or knockdown of NLRP3 can result in the suppression of Th17 differentiation [72]. Recently, NLRP12 has been recognized for its role as a checkpoint inhibitor of Th17 cell differentiation to serve as a negative regulator of inflammation and thereby regulate the severity of RA [73].

## NOD-like receptors in psoriatic arthritis

PsA is defined as a chronic and excessive inflammation that arises in various tissues such as the skin, entheses and joints [74]. This disease occurs in 0.05–0.25% of the general population and in 6–41% of psoriasis (PsO) patients [74]. PsA is also associated with an increased risk of mortality due to other comorbidities such as cardiovascular risk due to inflammation and metabolic alterations [13,74]. HLA class 1 alleles have been implicated in 35–50% of PsA and PsO heritability [13,74]. Pro-inflammatory cytokines such as IL-23, IL-17A and TNF have been implicated in PsA and PsO [74]. Wang and colleagues (2019) found that inhibiting the NLRP3 inflammasome with MCC950 resulted in reduced levels of IL-23R and decreased activation of the IL-23/IL-17 axis [75]. Juneblad and colleagues’ (2021) study is the first one to show that a genetic polymorphism in CARD8-C10X (rs2043211), an inflammasome-related gene, is associated with PsA [76]. Interestingly, there has been a debate about whether PsA and PsO are simply two presentations of the same disease as the underlying pathogenetic mechanisms are shared. Numerous genome-wide association studies have found that most genetic risk loci overlap between PsA and PsO [52]. This clinical overlap poses a challenge in measuring the risk of developing PsA in PsO patients [77]. Bowes and et al. (2015) found a genome-wide significant association (p = 1.49 × 10^−9^) of PTPN22 R620W (rs2476601) to PsA susceptibility; this association is PsA-specific and has an odds ratio of 1.32 [52,77]. At the time of this study, this was the fourth PsA-specific locus to be identified; identifying these loci are important to explore differences in underlying mechanisms and to create effective treatments.

Intriguingly, mutations in NLRC4 have been identified in autoinflammatory syndrome patients that have experienced urticarial-like skin rash and joint pain. These mutations cause hyperactivation of NLRC4 inflammasome and at least one patient was diagnosed with PsA [78]. Additional studies are needed to confirm the role of NLRC4 in driving PsA pathogenesis and investigate whether targeting this NLR member can lead to improved disease outcome.

## NOD-like receptors in spondylarthritis

Spondylarthritis is a chronic inflammatory disease, for which the cause is still unknown. Spondylarthritis is recognized as a group of diseases, that are usually identified simultaneously in a single patient or close family members [79]. This includes PsO, arthritis related to inflammatory bowel disease (IBD), JIA, reactive arthritis, and AS. The latter is used as the general prototype to model spondylarthritis [79]. The inflammatory cytokines IL-23 and IL-17 are critical to the pathogenesis of AS, where altered IL-23 signaling can cause abnormal IL-17 responses [80].

Investigating the role of NOD2 in IL-23 production emphasizes the interplay between the IL-23/IL-17 axis in AS [81]. PBMCs incubated with muramyl dipeptide (MDP) showed an upregulation of IL-23 and increased relative expression of NOD2 in AS samples compared to healthy controls, although the latter was not significant due to small sample size [81]. It is thought that the release of IL-23 can promote the release of IL-17 and lead to chronic inflammation, as seen in AS, although the trigger to release IL-23 is still unknown [81]. These results are interesting, as it was previously shown that NOD2 variants do not significantly affect the risk of developing AS, although it may play a role in susceptibility of the disease [82].

There are a multitude of studies probing the idea of certain genetic mutations and the susceptibility of AS [[83], [84], [85]]. For instance, HLA-B27 is a major genetic risk factor for AS [79,83]. There is a probable association between TNF receptor 1 signaling molecule (*TNFSF1A)*, TNFR -associated death domain (*TRADD)* protein, and interleukin-1 alpha *(IL-1α)*, and a definitive association with cytokine receptor interleukin-23 (*IL-23R)* and AS susceptibility [83]. There are still mixed results when looking at single nucleotide polymorphisms (SNPs), specifically in NLRP3. Genotyping AS patients revealed three *NLRP3* SNPs: rs35829419, rs4353135 and rs10733113, although none were associated with AS susceptibility [84]. Despite the large sample size this study was only done in a Swedish population [84]. Intriguingly, a later study revealed a significant correlation between *NLRP3* polymorphisms (rs4149570), *NLRP3* haplotypes and AS susceptibility [85].

Interestingly, inflammasome signaling seems to be upregulated in AS patients, where secreted IL-1β modulates IL-23 and Il-17 expression [86]. LPS-induced inflammasome activation increased IL-23 and IL-17 expression, which was suppressed with the addition of KCl (Potassium chloride, known to inhibit inflammasome activation) and reversed with IL-1β incubation [86]. The same phenomenon was observed by Kim et al. (2018) [87], where an increase in NLRP3, caspase-1, IL-1β, IL-23 and IL-17 was noted after LPS induction and suppressed by ascorbic acid treatment (shown to inhibit IL-1β expression in human 293T cells stimulated with MSU) [88]. An overexpression of inflammasome related genes, *NLRP3, CASP1 and IL-1β,* were observed in AS patient samples [86,87] and serum analysis of AS patients revealed higher levels of IL-1β and IL-18 inflammatory cytokines [86]. Although the activation of the inflammasome in AS is driven by inflammation, the exact trigger is unknown and requires further investigation.

Similar to other types of IA, dietary compounds that target NLR signaling have been studied in the context of AS. Resveratrol (3.4.5′-trihydroxy-trans-stilbene), a phytoalexin found in fruits, vegetables, chocolate, grapes, and wine, possesses anti-inflammatory properties [89]. Resveratrol slowed the progression and severity of AS in a proteoglycan induced AS mouse model. Mice gavaged with resveratrol exhibited slower progression and less severe AS. Mechanistically, resveratrol inhibited the TLR4/NF-κB/NLRP3 pathway, attenuated NLRP3 activation and cytokine production, and increased intestinal probiotic levels [89]. Furthermore, Micheliolide (MCL), a sesquiterpene lactone shown to help reduce the inflammatory response, decreased NLRP3, ASC, IL-1β, TNF-α, INF-γ, IL-6 and IL-18 protein expression in treated mice and increased IL- 4 [90].

There is still a lot of ambiguity surrounding the mechanism behind the inflammatory response in AS and additional studies investigating the role of NLR proteins in these diseases are needed to shed more light on disease pathogenesis.

## NOD-like receptors in juvenile idiopathic arthritis and juvenile spondylarthritis

JIA encompasses several heterogenous arthritis-related conditions that lead to chronic inflammation and disease onset for more than six weeks prior to the age of 16 years [14]. Interestingly, the role of PTPN22 1858 C/T allele (R620W variant) in JIA is akin to RA [52]. A meta-analysis of 4238 JIA patients and 6012 normal control subjects confirmed that this polymorphism is associated with JIA with an odds ratio of 1.311 [91]. A novel finding by Jørgensen and colleagues (2020) identified an extremely rare heterozygous missense variant in the CASP1 gene encoding pro-caspase-1 in a patient with systemic JIA (sJIA) and recurrent macrophage activation syndrome (MAS). This CASP1 variant was found to be gain-of-function in promoting inflammasome activation and NF-κB activation, ultimately leading to heightened levels of IL-1β, IL-6 and IL-18 production [92]. Recently, Wittmann and colleagues (2023) measured *ex vivo* inflammasome activation by detecting ASC speck formation in peripheral blood from oligoarticular and polyarticular JIA patients. The study discovered that both oligo- and poly-articular JIA patients had *ex vivo* inflammasome activation, albeit, more pronounced in oligo-articular JIA patients [93]. Patients with sJIA, a severe and life-threatening phenotype in the JIA group, have unusual cytokine patterns due to polymorphisms in several cytokine gene promoters [94]. Although no notable mutations in NLRP3 have been identified in sJIA patients, synovial fluid and sera contain elevated levels of IL-1β and IL-18, which are both dependent on NLRP3 inflammasome assembly and may indicate an increased activation of the inflammasome and enhanced caspase-1 activity [94]. Cader et al. (2016) explored the underlying mechanism between a SNP in *C13orf31* (LACC1) encoding p.C284R in a protein named fatty acid metabolism-immunity nexus (FAMIN) and its association with sJIA. Using ATP + LPS to activate the NLRP3 inflammasome, they found that M1 – classically activated – murine macrophages deficient for FAMIN resulted in low caspase-1 activity and IL-1β secretion. This suggests that the extent of NLRP3 inflammasome activation and IL-1β secretion is dictated by FAMIN [95]. Barnes and colleagues (2009) performed gene expression analysis on PBMCs from patients with JIA and found an upregulation of several pathways in the innate immune system along with complement and coagulation pathways [96]. Interestingly, Brown and colleagues (2018) performed whole transcriptome analysis and identified 214 differentially expressed genes in neutrophils from children with active sJIA and intermediate expression in patients with clinically inactive disease. Of these pathways, AIM2, IL18RAP and NLRC4 were significantly upregulated [97]. However, a direct link between NLRC4 and sJIA pathogenesis remains to be explored.

Juvenile spondyloarthritis (JSpA) encompasses chronic and heterogenous seronegative inflammatory disorders and is associated with the HLA-B27 genotype [98,99]. Compared to adults, JSpA has been associated with lower HLA-B27 positivity, less peripheral arthritis and enthesitis and instead has more hip arthritis, axial involvement and acute anterior uveitis [99]. Reduced expression of NLRP3 has been observed in JSpA and it is proposed that this contributes to permeability of the gut and subclinical inflammation which is seen in studies with defective NLRP3 inflammasome signaling (i.e., inflammatory bowel disease) [98,99]. On the other hand, it is important to note that a population study in Croatia found no association between a SNP in NLRP3 (Q705K) with JSpA or JIA [100].

## Future perspectives

**Fig. 1:**
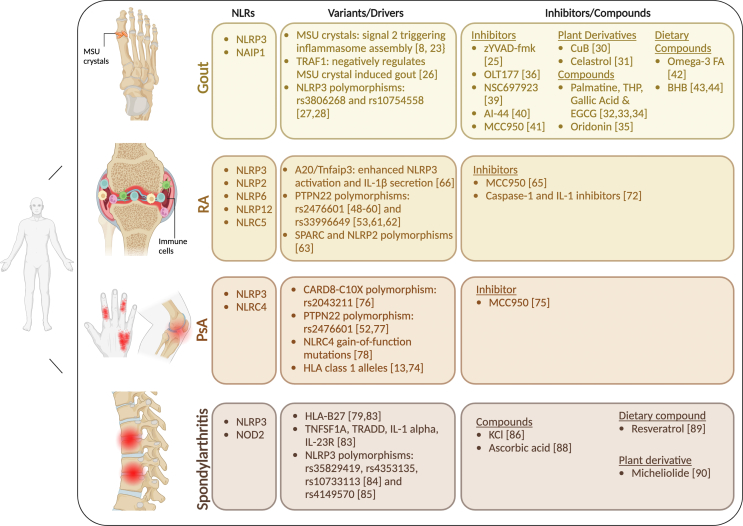
**Contributions of** NOD-like receptors (**NLRs)to Inflammatory arthritis** (IA)**.** Several NLR family members contribute to the pathogenesis and development of IA. Polymorphisms in these NLR proteins have also been shown to be associated with risk to various IA diseases. Studies have also investigated inhibitors and other compounds (synthetic, natural and dietary) to validate the role of NLRs in driving these diseases and as potential therapies. *Figure was created with*BioRender.com.

The studies discussed in this review demonstrate that major advances have been made in understanding how NLRs contribute to the development of IA [Fig. 1]. The vast majority of these studies have investigated the involvement of NLRP3 in IA owing its well-defined role in inflammasome activation and production of the potent pro-inflammatory cytokine, IL-1β. Despite the predominant role that innate immune cells and inflammatory signaling pathways play in pathogenesis of IA, the role of other NLR members in these diseases is understudied. Therefore, future research should fill these gaps, especially in NLRs that are currently implicated in autoinflammatory disease. These may include NLRX1, which has been shown to play a key role in regulating mitophagy and innate immune signaling pathways, and NOD1 and 2, which are associated with systemic inflammatory diseases.

Dietary, natural, and synthetic compounds that target NLRP3 have been the focus of research into development of novel therapeutics for IA diseases. However, therapies targeting other NLR proteins are still lacking and may represent an important area of development in the future.

## Funding

Our work in this field is supported by grants 201809PJT from the 10.13039/501100000024Canadian Institutes of Health Research, STAR-18-0276 from the 10.13039/501100000142Arthritis Society, and 100066519 from The Krembil Foundation.

## Conflicts of interest

The authors have declared that no competing interests exist.
